# Apigenin Provides Structural Protection to Human Fibrinogen against Nitrosative Stress: Biochemical and Molecular Insights

**DOI:** 10.3390/biom14050576

**Published:** 2024-05-13

**Authors:** Aisha Farhana, Abdullah Alsrhani, Yusuf Saleem Khan, Mohammad Salahuddin, Mohammed Ubaidullah Sayeed, Zafar Rasheed

**Affiliations:** 1 Department of Clinical Laboratory Sciences, College of Applied Medical Sciences, Jouf University, Sakaka 72388, Aljouf Province, Saudi Arabia; afalserhani@ju.edu.sa; 2 Department of Anatomy, College of Medicine, University of Hail, Hail 55476, Hail Province, Saudi Arabia; 3 Department of Physiology, College of Medicine, Jouf University, Sakaka 72388, Aljouf Province, Saudi Arabia; mskhaja@ju.edu.sa; 4 Department of Pathology, College of Medicine, Jouf University, Sakaka 72388, Aljouf Province, Saudi Arabia; usmohammed@ju.edu.sa; 5 Department of Pathology, College of Medicine, Qassim University, Buraidah 51452, Qassim Province, Saudi Arabia; zafarrasheed@qu.edu.sa

**Keywords:** apigenin, peroxynitrite, fibrinogen, biochemical studies, oxidative damage, nitrosative stress

## Abstract

Background: Peroxynitrite (ONOO^−^) is an oxidant linked with several human pathologies. Apigenin, a natural flavonoid known for its health benefits, remains unexplored in relation to ONOO^−^ effects. This study investigated the potential of apigenin to structurally protect fibrinogen, an essential blood clotting factor, from ONOO^−^-induced damage. Methods: Multi-approach analyses were carried out where fibrinogen was exposed to ONOO^−^ generation while testing the efficacy of apigenin. The role of apigenin against ONOO^−^-induced modifications in fibrinogen was investigated using UV spectroscopy, tryptophan or tyrosine fluorescence, protein hydrophobicity, carbonylation, and electrophoretic analyses. Results: The findings demonstrate that apigenin significantly inhibits ONOO^−^-induced oxidative damage in fibrinogen. ONOO^−^ caused reduced UV absorption, which was reversed by apigenin treatment. Moreover, ONOO^−^ diminished tryptophan and tyrosine fluorescence, which was effectively restored by apigenin treatment. Apigenin also reduced the hydrophobicity of ONOO^−^-damaged fibrinogen. Moreover, apigenin exhibited protective effects against ONOO^−^-induced protein carbonylation. SDS-PAGE analyses revealed that ONOO^−^treatment eliminated bands corresponding to fibrinogen polypeptide chains Aα and γ, while apigenin preserved these changes. Conclusions: This study highlights, for the first time, the role of apigenin in structural protection of human fibrinogen against peroxynitrite-induced nitrosative damage. Our data indicate that apigenin offers structural protection to all three polypeptide chains (Aα, Bβ, and γ) of human fibrinogen. Specifically, apigenin prevents the dislocation or breakdown of the amino acids tryptophan, tyrosine, lysine, arginine, proline, and threonine and also prevents the exposure of hydrophobic sites in fibrinogen induced by ONOO^−^.

## 1. Introduction

Fibrinogen is a crucial protein, which plays an important role in blood clotting. It is produced in the liver and circulates in the blood plasma. When a blood vessel is damaged, a series of biochemical reactions known as the coagulation cascade is initiated. Fibrinogen is crucial to the last step of this cascade, converting into fibrin, which forms a sticky protein meshwork that acts as the framework for a blood clot. The clot serves as a plug to prevent excessive bleeding and promotes wound healing [1,2] Fibrinogen plays a crucial role in tissue regeneration by providing mechanical support for the migration and proliferation of cells. It helps in maintaining the integrity of blood vessels [1,3]. Alterations in fibrinogen levels or its structural characteristics can disrupt the delicate balance of hemostasis, leading to thrombotic events, such as deep vein thrombosis or arterial clot formation, as well as bleeding disorders [3,4]. It is worth noting that fibrinogen also performs its protective function against inflammation. During tissue injury or inflammation, circulating fibrinogen interacts with inflammatory cells and mediators, acting as an adhesive molecule that assists in recruiting immune cells to the site of injury or infection [5]. It is crucial to recognize that changes in fibrinogen structures, levels, or functional properties are connected to various pathological conditions [6,7]. Elevated fibrinogen levels have been observed in acute and chronic inflammatory diseases, cardiovascular disorders, and specific types of cancer. Imbalances in fibrinogen levels or alterations in its structure can result in clotting disorders or heightened vulnerability to bleeding, and they have been linked to various inflammatory diseases, cardiovascular disorders, and certain cancers [4,6,7]. In addition, fibrinogen measurements are valuable diagnostic and prognostic indicators for assessing the risk of thrombotic events and monitoring disease progression [8,9]. Therefore, understanding the significance of fibrinogen is vital in the fields of hematology, cardiovascular medicine, molecular immunology, and inflammation.

Peroxynitrite is an extremely reactive compound consisting of oxygen and nitrogen atoms categorized as a reactive oxygen and nitrogen species (RONS) and represented by the chemical formula ONOO^−^. It exhibits strong oxidizing and nitrating properties, leading to damage in biomolecules across various biological systems [10]. Peroxynitrite is produced in humans in response to various internal and external stressors, including toxins, UV light, and stressors, in different pathological scenarios [11]. It is widely accepted that the harmful effects of nitric oxide primarily arise from peroxynitrite, which has high reactivity and reacts readily with different biomolecules like proteins, lipids, and nucleic acids [12,13,14]. Apart from causing oxidative harm to biomolecules, peroxynitrite also triggers several cell-signaling pathways leading to oxidative injury and cell death through necrosis or apoptosis [15,16]. Moreover, peroxynitrite is implicated in the aberrant activation of molecular pathways associated with numerous inflammatory and neurodegenerative conditions, and cancer [15,16]. Furthermore, peroxynitrite, along with other reactive nitrogen species (RNS), is involved in initiating and advancing autoimmune responses [17,18]. Apigenin is a naturally occurring flavonoid found in various fruits, vegetables, and herbs. It belongs to the flavone subclass of flavonoids and is recognized for its unique chemical structure and diverse biological activities [19]. Chemically known as 4′,5,7-trihydroxyflavone, apigenin is a yellow crystalline solid with the molecular formula C_15_H_10_O_5_ (PubChem CID: 5280443). Its distinctive features include two aromatic rings connected by a central three-carbon bridge, as well as hydroxyl groups at positions 4′, 5, and 7 of the C-ring, which contribute to its unique biological properties [20]. Notably, apigenin acts as a potent antioxidant by scavenging free radicals and inhibiting oxidative stress. It suppresses the formation of oxidative radicals and enhances the activity of endogenous antioxidant enzymes [21,22]. However, despite its known antioxidant properties, the effects of apigenin on ONOO^−^-induced oxidative damage to fibrinogen have not yet been investigated.

The study was conducted to examine the potential protective effect of apigenin on structural damage to human fibrinogen caused by peroxynitrite. Our findings provide novel insights into the potential therapeutic uses of apigenin in treating disorders associated with ONOO^−^-induced damage. This novel information may hold significant implications for the development of innovative therapeutic approaches targeting ONOO^−^-related human metabolic and inflammatory pathological conditions.

## 2. Materials and Methods

### 2.1. Preparation of Human Fibrinogen and Apigenin Solutions

Human fibrinogen was procured from Sigma-Aldrich (Saint Louis, MO, USA) and further purified through ammonium sulfate precipitation (up to 25%) followed by centrifugation and suspension in PBS (10 mM, pH 7.4). Apigenin (4′,5,7-trihydroxyflavone) was purchased from R&D Systems (Minneapolis, MN, USA) and suspended in dimethyl sulfoxide to prepare 100 mM of stock. A working solution of apigenin was diluted in PBS (10 mM, pH 7.4).

### 2.2. Treatment of Fibrinogen UsingApigenin and Peroxynitrite

Human fibrinogen was subjected to simultaneous treatment with apigenin and peroxynitrite anion (ONOO^−^) in PBS (10 mM, pH 7.4) following the methodology described by Rasheed et al., 2018 [12]. Briefly, human fibrinogen at a concentration of 2.9412 µM was treated with apigenin at varying concentrations (50–100 µM). The treatment also involved a mixture containing sodium nitroprusside (0.5 mM; Sigma, MO, USA), pyrogallol (0.5 mM; Sigma, MO, USA), and diethylenetriaminepentaacetic acid (0.5 mM; Sigma, MO, USA), which was carried out at 37 °C for 24 h. To remove excess salts, the reaction mixture was dialyzed against PBS. Negative controls consisted of fibrinogen without apigenin, sodium nitroprusside, or pyrogallol (referred to as native fibrinogen), while positive controls included fibrinogen without apigenin but with sodium nitroprusside and pyrogallol (referred to as Fibrinogen-ONOO^−^).

### 2.3. Ultraviolet Spectroscopy

The UV absorption spectra of untreated and treated fibrinogen protein samples were assessed using the PerkinElmer Spectrophotometer (PerkinElmer Ltd., Beaconsfield, UK). The calculation of hypochromicity percentage at 280 nm was carried out following the methodology outlined by Rasheed et al., 2022 [23].

% Hypochromicity = [Native fibrinogen OD_280 nm_ − Modified fibrinogen OD_280nm_)/Native fibrinogen OD_280 nm_] × 100

### 2.4. Tryptophan and Tyrosine Fluorescence Emission Studies

Alterations in tryptophan or tyrosine–tryptophan amino acid residues were examined by exciting the protein samples at 295 nm and 280 nm, respectively, following the previously described method by Möller and Denicola, 2022 [24]. The fluorescence intensities (FI) were measured using a plate reader (AnthosZenyth, Salzburg, Austria). The percentage change in FI was determined using the previously described equation by Rasheed et al., 2006 [25].

% Reduce of FI = [FI_untreated fibrinogen_ − FI_modified fibrinogen_)/FI _untreated fibrinogen_] × 100

or

% Increase in FI = [FI_modified fibrinogen_− FI _untreated fibrinogen_)/FI_modified fibrinogen_] × 100

### 2.5. bis-ANS-Binding Fluorescence Studies

The protein hydrophobicity was studied by probing with *bis*-ANS-binding fluorescence in all protein samples of fibrinogen, as described previously [26]. The ANS–fibrinogen complexes were excited at 380 nm, and FI were quantified as in a published procedure [27].

### 2.6. SDS-PAGE

The treated and untreated fibrinogen samples (35 µg) along with the Life Technologies Precision Plus Protein Markers (Carlsbad, CA, USA) were subjected to electrophoresis. To observe all three fibrinogen chains, all fibrinogen protein samples were denatured through SDS, and its inter-chain disulfide bonds were reduced using β-mercaptoethanol. The electrophoresis was conducted using a 10% resolving gel with a 2.5% stacking with an applied voltage of 80 V. The gel was visualized using Coomassie Brilliant Blue R-250 (Sigma-Aldrich, Merck Group, St. Louis, MO, USA).

### 2.7. Protein Carbonyl Content Estimation

The levels of oxidation in fibrinogen protein samples were assessed using the Protein Carbonyls Colorimetric Assays Kit (Cayman Chemical, Ann Arbor, MI, USA).

## 3. Results

**Figure 1:**
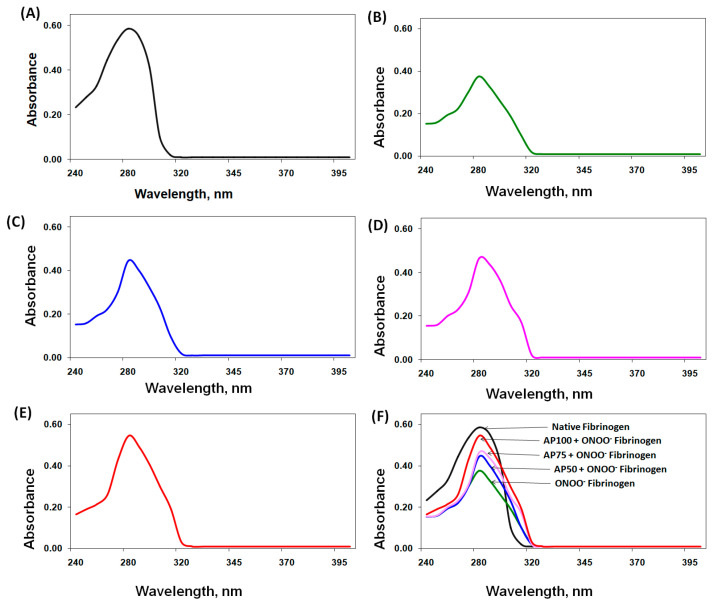
Ultraviolet (UV) absorption spectroscopic studies. (**A**) UV absorption spectrum of native fibrinogen. (**B**) UV absorption spectrum of peroxynitrite (ONOO^−^)-treated fibrinogen (ONOO^−^-fibrinogen). (**C**) UV absorption spectrum of apigenin (AP, 50 µM) + ONOO^−^-fibrinogen. (**D**) UV absorption spectrum of apigenin (AP, 75 µM) + ONOO^−^-fibrinogen. (**E**) UV absorption spectrum of apigenin (AP, 100 µM) + ONOO^−^-fibrinogen. (**F**) Overlapping of all studied fibrinogen protein samples. The concentrations of all protein samples were 1.47 µM, dialyzed using 10 mM PBS (pH = 7.0).

Human fibrinogen underwent treatment with ONOO^−^ and apigenin, and the resulting changes were analyzed. The UV spectral studies of ONOO^−^-treated fibrinogen showed a 35.7% decrease in absorption intensity at 280 nm, indicating hypochromicity (Figure 1A,B). Importantly, the addition of apigenin (50–100 µM) to the protein reaction solution significantly reduced the hypochromicity induced by ONOO^−^ (*p* < 0.05). These results are given in Figure 1C–E. The role of apigenin against ONOO^−^-induced impairment to fibrinogen was demonstrated by overlapping of all samples of UV spectra (Figure 1F). The degree of protection provided by different doses of apigenin was quantified by comparing the hypochromicity of each protein sample. The results showed that apigenin at 50 µM provided 32.8% protection against peroxynitrite-induced hypochromicity at 280 nm, while higher doses of apigenin (75 µM and 100 µM) offered increased protection levels of 42.6% and 80.9%, respectively (Table 1).

**Figure 2:**
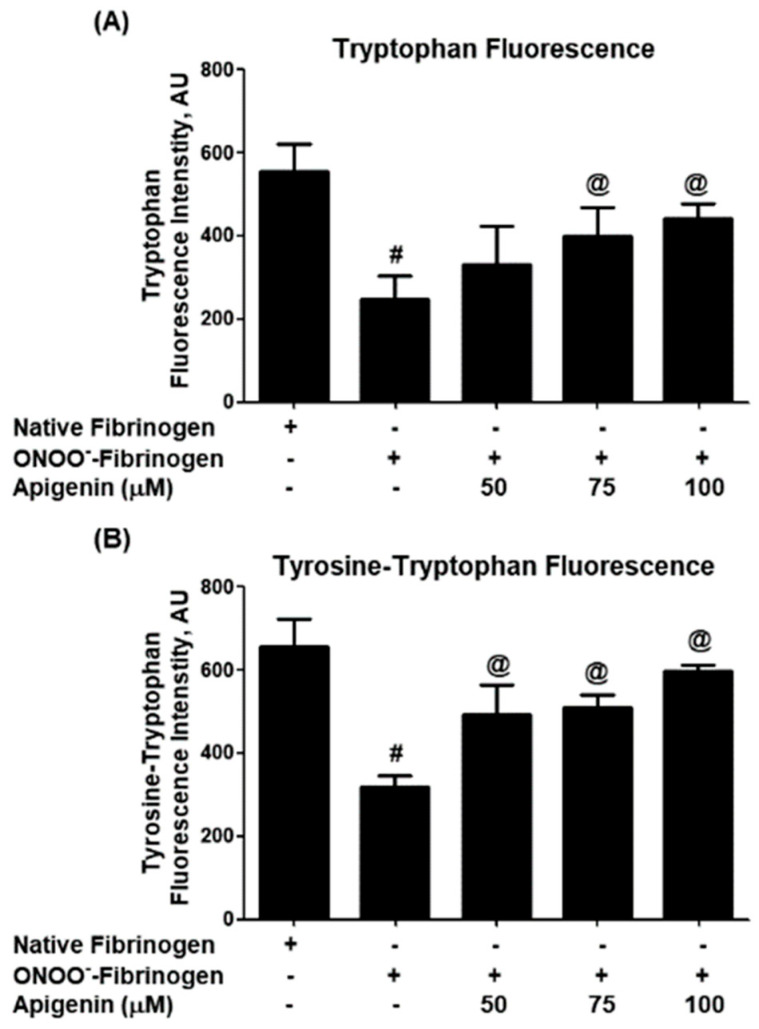
Tryptophan and tyrosine fluorimetry. (**A**) Effect of apigenin on peroxynitrite (ONOO^−^)-induced alterations in the tryptophan residues of fibrinogen protein. The tryptophan residues were excited at a wavelength of 295 nm. # *p* < 0.001 versus native fibrinogen; @ *p* < 0.05 versus #. (**B**) Effect of apigenin on peroxynitrite (ONOO^−^)-induced alterations in the tyrosine–tryptophan residues of fibrinogen protein. The tyrosine and tryptophan residues were excited at a wavelength of 280 nm. # *p* < 0.001 versus native fibrinogen; @ *p* < 0.05 versus #.

Further investigation of the structural protection provided by apigenin against ONOO^−^ was carried out using tryptophan fluorescence excitation studies. When fibrinogen was treated with ONOO^−^ alone, a significant decrease in tryptophan fluorescence intensity was observed (*p* < 0.001; Figure 2A). However, the addition of apigenin significantly enhanced tryptophan fluorescence intensity (*p* < 0.05). Similar alterations in the fluorescence intensity of both tryptophan and tyrosine residues were observed when fibrinogen samples were excited at 280 nm (Figure 2B). The percentage of protection of tryptophan residues provided by different doses of apigenin was determined, showing that apigenin at 50 µM offered 27.3% protection against ONOO^−^-induced damage, with higher doses of apigenin (75 µM and 100 µM) providing 49.0% and 54.2% protection, respectively (A) in Table 2. Similar protection percentages were observed for the tyrosine–tryptophan residues (B) in Table 2.

**Figure 3:**
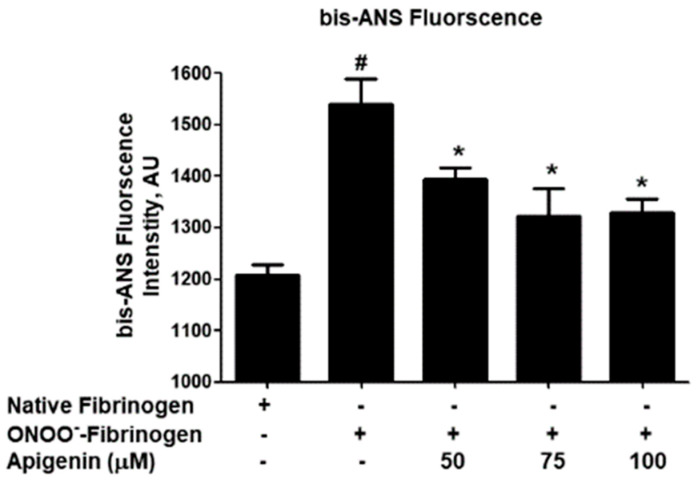
Hydrophobic patches detection. Effect of apigenin on peroxynitrite (ONOO^−^)-induced hydrophobic patches on fibrinogen protein. The fibrinogen samples were excited at 380 nm. # *p* < 0.001 versus native fibrinogen; * *p* < 0.05 versus #.

The role of apigenin against ONOO^−^-induced alterations in hydrophobic sites was investigated using the hydrophobic probe bis-ANS. Peroxynitrite significantly enhanced fibrinogen hydrophobicity (*p* < 0.001; Figure 3), but the addition of apigenin reduced the hydrophobicity of the protein (*p* < 0.01). The percentage of safety provided by apigenin against ONOO^−^-induced hydrophobicity changes was determined, with apigenin at 50 µM offering 38.7% protection and higher doses of apigenin (75 µM and 100 µM) providing 58.5% and 61.3% protection, respectively (Table 3).

**Figure 4:**
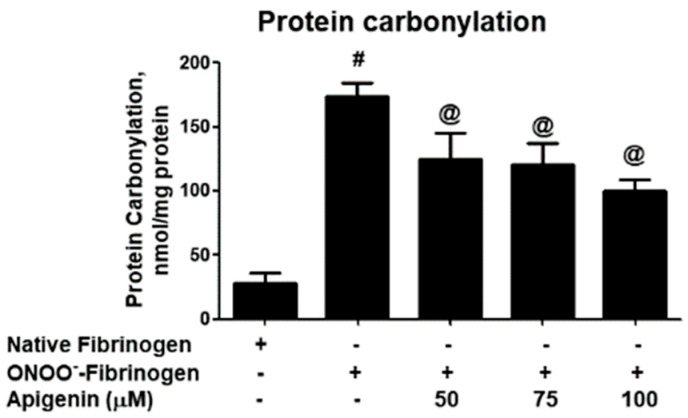
Protein carbonyl group formation. Effect of apigenin on peroxynitrite (ONOO^−^)-induced protein carbonyl group formation in fibrinogen protein. Protein carbonyl contents in native fibrinogen, ONOO^−^-modified fibrinogen, and apigenin-treated ONOO^−^-fibrinogen. # *p* < 0.0001 versus native fibrinogen; @ *p* < 0.05 versus #.

**Figure 5:**
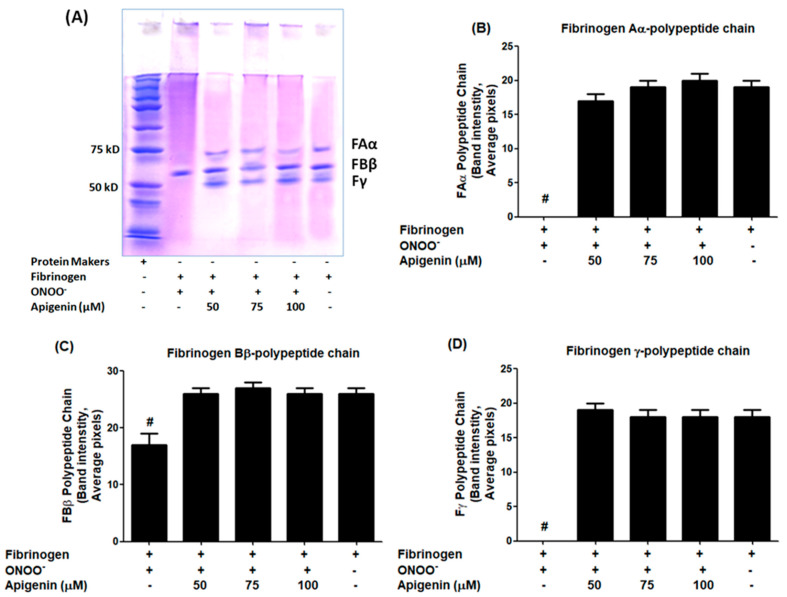
SDS-PAGE for the detection of fibrinogen in subunits. (**A**) SDS-PAGE of fibrinogen, ONOO^−^-damaged fibrinogen, and apigenin-treated ONOO^−^-fibrinogen. Band intensities of fibrinogen alpha (FAα) polypeptide chain (**B**), fibrinogen beta (FAβ) polypeptide chain (**C**), and fibrinogen alpha (Fγ) polypeptide chain (**D**) were measured using the Un-Scan-It software, Window Version-7.1, and presented from the four independent pixel readings. # *p* < 0.001 versus native or apigenin-treated fibrinogen samples. Original images of (**A**) can be found in Figure S1.

To evaluate the role of apigenin, the protein carbonyl contents were analyzed (Figure 4). The data revealed a significant rise in protein carbonyl formation in fibrinogen treated with peroxynitrite alone (*p* < 0.001). However, the addition of apigenin resulted in a remarkable decrease in protein carbonyl formation (*p* < 0.05) (Figure 4). The percentage of protection provided by different doses of apigenin against protein carbonylation was determined, and it is summarized in Table 4. Moreover, the therapeutic potential of apigenin was evaluated using SDS-PAGE (Figure 5A). The untreated fibrinogen sample exhibited three distinct bands corresponding to the three polypeptide chains FAα, FBβ, and Fγ. However, upon treatment of fibrinogen with peroxynitrite, the bands representing FAα and Fγ chains completely disappeared (Figure 5B,D), while the band intensity for the FAα chain was significantly reduced, compared to the intensity at the beginning of the gel (*p* < 0.05; Figure 5C), indicating it acquires altered mobility after chemical modification by peroxynitrite.In contrast, the addition of apigenin to the reaction mixture resulted in almost similar band intensities for all three polypeptides of fibrinogen, as seen in the untreated fibrinogen (*p* > 0.05; Figure 5A–D).

## 4. Discussion

This research presents the novel finding of apigenin’s protective effect on fibrinogen protein structure against peroxynitrite-induced nitrosative damage. Nitric oxide’s harmful effects are primarily linked to peroxynitrite formation, which damages biomolecules such as proteins, nucleic acids, and lipids, initiating oxidative structural harm and a chain reaction of free radical generation [10,16,28,29]. Peroxynitrite is recognized as one of the most detrimental free radicals due to its lethal effects. Apigenin, a natural compound found in plants, has garnered attention for its various health-promoting properties, including antioxidant, anti-inflammatory, anticancer, antimicrobial, and neuroprotective effects [19,20,29,30,31,32]. It has been shown to modulate redox homeostasis and regulate autophagy, further contributing to its anti-inflammatory effects [33]. Flavonoids, like apigenin, have been reported to reduce oxidative stress and inflammatory cytokines, potentially preventing cardiac injury from endotoxins [34,35,36,37]. Consequently, apigenin has become a subject of significant interest for its potential theraputic applications, particularly in preventing various human disorders, including cancer and cardiovascular diseases, where peroxynitrite plays a pivotal role.

Abnormality in fibrinogen function caused the inappropriate formation of fibrin clots, which can lead to arterial and venous thrombotic disorders, which can be life-threatening [4]. Thrombotic disorders often occur in the presence of inflammation and oxidative stress. Fibrinogen is susceptible to oxidative modifications, which can impact its structure and function and have been associated with disease development [38,39]. Oxidative modifications of fibrinogen can cause structural changes that impair its ability to form fibrin. [39]. However, the specific effects of peroxynitrite-induced damage on fibrinogen structure and the apigenin role e against such damage have not yet been explored. For the first time, we have discovered that apigenin has the potential to counteract peroxynitrite-induced damage to fibrinogen in an invitro setting.

Our experiments involved treating human fibrinogen with both peroxynitrite and apigenin and analyzing the structural changes using various techniques. We used UV absorption spectroscopy to examine the alterations in the structure of fibrinogen. The findings showed that peroxynitrite caused significant damage, as evidenced by a decrease in absorption at 280 nm, which indicates changes in aromatic amino acids. However, the addition of apigenin protected against these oxidative modifications induced by peroxynitrite. Similar protective effects of apigenin were observed through tryptophan and tyrosine fluorescence studies. Peroxynitrite-induced damage led to reduced fluorescence intensity, indicating structural changes in fibrinogen. However, the presence of apigenin increased the fluorescence intensity of tryptophan and tyrosine residues, indicating protection against nitrosative damage.

To further investigate the potential of apigenin in preventing peroxynitrite-induced damage, we conducted bis-ANS-binding fluorimetry studies. By assessing the fluorescence emitted by the hydrophobic probe bis-ANS, we can infer the presence of exposed hydrophobic patches. Our data revealed that apigenin treatment significantly inhibited peroxynitrite-induced hydrophobicity of fibrinogen, indicating its ability to prevent structural alterations in these hydrophobic sites. These findings demonstrate that apigenin has a protective effect against peroxynitrite-induced damage to fibrinogen. This protective effect was observed through the prevention of structural modifications, the preservation of tryptophan and tyrosine residues, and the inhibition of peroxynitrite-induced hydrophobicity. Protein carbonylation refers to the non-enzymatic irreversible oxidative modifications that occurring in proteins as a manifestation of oxidative stress. These modifications primarily target amino acid residues, such as lysine, arginine, proline, and threonine [38,40,41,42]. The measurement of carbonyl contents has become a reliable biomarker for assessing oxidative damage to proteins in various disorders [40,42,43]. Therefore, we analyzed the protein carbonyl formation in both untreated and treated fibrinogen samples in this study. Our novel findings indicate a noteworthy rise in protein carbonyl formation in fibrinogen treated with peroxynitrite, as opposed to the untreated protein. Importantly, the inclusion of apigenin in the reaction mixture successfully reversed this increase in carbonyl formation. These results strongly suggest that apigenin provides protection to lysine, arginine, proline, and threonine residues in human fibrinogen, effectively guarding against peroxynitrite-induced damage.

Human fibrinogen has three pairs of polypeptide chains, namely Aα, Bβ, and γ, with 66.5 kDa, 52 kDa, and 46.5 kDa, respectively [44]. To assess the impact of peroxynitrite-induced damage on fibrinogen and the protective potential of apigenin on these polypeptide chains, we performed an SDS-PAGE analysis on all fibrinogen samples. Untreated fibrinogen samples exhibited three distinct bands on SDS-PAGE, ranging from 50–75 kDa, corresponding to the three pairs of polypeptide chains Aα, Bβ, and γ, which aligns with previous studies [44]. However, upon treatment with peroxynitrite, the bands for the Aα and γ polypeptide chains completely disappeared, indicating severe structural damage caused by these peroxynitrite. Importantly, the addition of apigenin to the fibrinogen samples resulted in the reappearance of all three bands, representing the three polypeptide chains of fibrinogen observed in the untreated samples. This strongly suggests that apigenin offers structural protection to human fibrinogen against peroxynitrite generation. These findings present new evidences that apigenin is a strong inhibitor of peroxynitrite-induced dent in human fibrinogen, preserving the integrity of the three polypeptide chains.

## 5. Conclusions

This study demonstrates the potential of apigenin in protecting human fibrinogen against nitrosative damage induced by peroxynitrite. The novel findings highlight that apigenin offers structural protection to all three polypeptide chains of human fibrinogen: Aα, Bβ, and γ. Furthermore, our data provide evidence that apigenin safeguards the integrity of key amino acids, including tryptophan, tyrosine, lysine, arginine, proline, and threonine, and provides protection of hydrophobic sites within the protein structure of fibrinogen, preventing peroxynitrite-induced oxidative damage. These results are of great significance and hold promise for developing novel therapeutic strategies aimed at managing disorders where peroxynitrite plays a pivotal role. The protective effects of apigenin on human fibrinogen may open new avenues for targeted interventions to alleviate the detrimental effects of peroxynitrite and preserve the structural integrity of fibrinogen in various pathological conditions.

## Figures and Tables

**Table 1:** Protective effect of apigenin on peroxynitrite-induced hypochromic alterations in aromatic amino acids of fibrinogen.

SN.	Comparative Assessment of Hypochromicity	Alteration at λmax (Hypo or Hyper-Chromicity)	% Protection on Hypochromicity by AP
1.	Native Fibrinogen v/s ONOO^−^-Fibrinogen	35.7% hypochromicity	-
2.	AP50-ONOO^−^-Fibrinogen v/s Native Fibrinogen	23.9% hypochromicity	32.8
3.	AP50-ONOO^−^-Fibrinogen v/s ONOO^−^-Fibrinogen	6.9% hyperchromicity	-
4.	AP75-ONOO^−^-Fibrinogen v/s Native Fibrinogen	20.5% hypochromicity	42.6
5.	AP75-ONOO^−^-Fibrinogen v/s ONOO^v^-Fibrinogen	8.9% hyperchromicity	-
6.	AP100-ONOO^−^-Fibrinogen v/s Native Fibrinogen	6.8% hypochromicity	80.9
7.	AP100-ONOO^−^-Fibrinogen v/s ONOO^v^-Fibrinogen	31.0% hyperchromicity	-

**Abbreviation:** ONOO^−^, peroxynitrite; AP50, Apigenin at 50 µM; AP75, Apigenin at 75 µM; AP100, Apigenin at 100 µM; AP, Apigenin.

**Table 2:** (A) Protective effect of apigenin on peroxynitrite-induced alterations in tryptophan residues of fibrinogen. (B) Protective effect of apigenin on peroxynitrite-induced alterations in tyrosine and tryptophan residues of fibrinogen.

A. Protective Effect of Apigenin on Peroxynitrite-Induced Alterations in Tryptophan Residues of Fibrinogen.			
SN.	Comparative Assessment of Trp Residues	Trp-Fluorescence Alterations	% Protection by AP
1.	Native Fibrinogen v/s ONOO^−^-Fibrinogen	55.3% FI. decreased	-
2.	AP50-ONOO^−^-Fibrinogen v/s Native Fibrinogen	40.2% FI. decreased	27.3
3.	AP50-ONOO^−^-Fibrinogen v/s ONOO^−^-Fibrinogen	25.2% FI. increased	-
4.	AP75-ONOO^−^-Fibrinogen v/s Native Fibrinogen	28.2% FI. decreased	49.0
5.	AP75-ONOO^−^-Fibrinogen v/s ONOO^−^-Fibrinogen	37.7% FI. increased	-
6.	AP100-ONOO^−^-Fibrinogen v/s Native Fibrinogen	25.3% FI. decreased	54.2
7.	AP100-ONOO^−^-Fibrinogen v/s ONOO^−^-Fibrinogen	44.0% FI. increased	-
**B.** **Protective Effect of Apigenin on Peroxynitrite-Induced Alterations in Tyrosine and Tryptophan Residues of Fibrinogen**			
**SN.**	**Comparative Assessment of Tyr-Trp Residues**	**Tyr-Trp-Fluorescence Alterations**	**% Protection by AP**
1.	Native Fibrinogen v/s ONOO^−^-Fibrinogen	51.6% FI. decreased	-
2.	AP50-ONOO^−^-Fibrinogen v/s Native Fibrinogen	24.8% FI. decreased	50.9
3.	AP50-ONOO^−^-Fibrinogen v/s ONOO^−^-Fibrinogen	35.6% FI. increased	-
4.	AP75-ONOO^−^-Fibrinogen v/s Native Fibrinogen	22.4% FI. decreased	56.6
5.	AP75-ONOO^−^-Fibrinogen v/s ONOO^−^-Fibrinogen	37.6% FI. increased	-
6.	AP100-ONOO^−^-Fibrinogen v/s Native Fibrinogen	9.8% FI. decreased	81.0
7.	AP100-ONOO^−^-Fibrinogen v/s ONOO^−^-Fibrinogen	46.7% FI. increased	-

**Abbreviation:** FI, fluorescence intensity; ONOO^−^, peroxynitrite; AP50, Apigenin at 50 µM; AP75, Apigenin at 75 µM; AP100, Apigenin at 100 µM; AP, Apigenin; Trp, tryptophan; Tyr, tyrosine.

**Table 3:** Protective effect of apigenin on peroxynitrite-induced hydrophobic patches in fibrinogen.

SN.	Comparative Assessment of Hydrophobicity	bis-ANS Binding Fluorescence	% Hydrophobic Protection by AP
1.	Native Fibrinogen v/s ONOO^−^-Fibrinogen	21.2% FI. increased	-
2.	AP50-ONOO^−^-Fibrinogen v/s Native Fibrinogen	13.0% FI. increased	38.7
3.	AP50-ONOO^−^-Fibrinogen v/s ONOO^−^-Fibrinogen	9.3% FI. decreased	-
4.	AP75-ONOO^−^-Fibrinogen v/s Native Fibrinogen	8.8% FI. increased	58.5
5.	AP75-ONOO^−^-Fibrinogen v/s ONOO^−^-Fibrinogen	13.6% FI. decreased	-
6.	AP100-ONOO^−^-Fibrinogen v/s Native Fibrinogen	8.2% FI. increased	61.3
7.	AP100-ONOO^−^-Fibrinogen v/s ONOO^−^-Fibrinogen	14.1% FI. decreased	-

**Abbreviation:** bis-ANS, 4,4′-dianilino-1,1′-binaphthyl-5,5′-disulfonic acid; FI, fluorescence intensity; ONOO^−^, peroxynitrite; AP50, Apigenin at 50 µM; AP75, Apigenin at 75 µM; AP100, Apigenin at 100 µM; AP, apigenin.

**Table 4:** Protective effect of apigenin on peroxynitrite-induced protein carbonylation in fibrinogen.

SN.	Comparative Assessment of Carbonylation	Alterations in Carbonyl Formation	% Protection of Carbonylation by AP
1.	Native Fibrinogen v/s ONOO^−^-Fibrinogen	83.6% increased	-
2.	AP50-ONOO^−^-Fibrinogen v/s Native Fibrinogen	77.2% increased	7.6
3.	AP50-ONOO^−^-Fibrinogen v/s ONOO^−^-Fibrinogen	28.1% decreased	-
4.	AP75-ONOO^−^-Fibrinogen v/s Native Fibrinogen	76.3% increased	8.7
5.	AP75-ONOO^−^-Fibrinogen v/s ONOO^−^-Fibrinogen	30.9% decreased	-
6.	AP100-ONOO^−^-Fibrinogen v/s Native Fibrinogen	71.5% increased	14.5
7.	AP100-ONOO^−^-Fibrinogen v/s ONOO^−^-Fibrinogen	42.5% decreased	-

**Abbreviation:** ONOO^−^, peroxynitrite; AP50, Apigenin at 50 µM; AP75, Apigenin at 75 µM; AP100, Apigenin at 100 µM; AP, Apigenin.

## Data Availability

All data and materials used in this study are available from the corresponding author and will be provided upon reasonable request.

## Supplementary Materials

The following supporting information can be downloaded at: https://www.mdpi.com/article/10.3390/biom14050576/s1, Figure S1: Original images of Figure 5A.
