# Supplementary figures and images for: Apigenin Provides Structural Protection to Human Fibrinogen against Nitrosative Stress: Biochemical and Molecular Insights
Source: Biomolecules. 2024 May 13;14(5):576. doi: 10.3390/biom14050576 (PMC11117476; doi:10.3390/biom14050576)

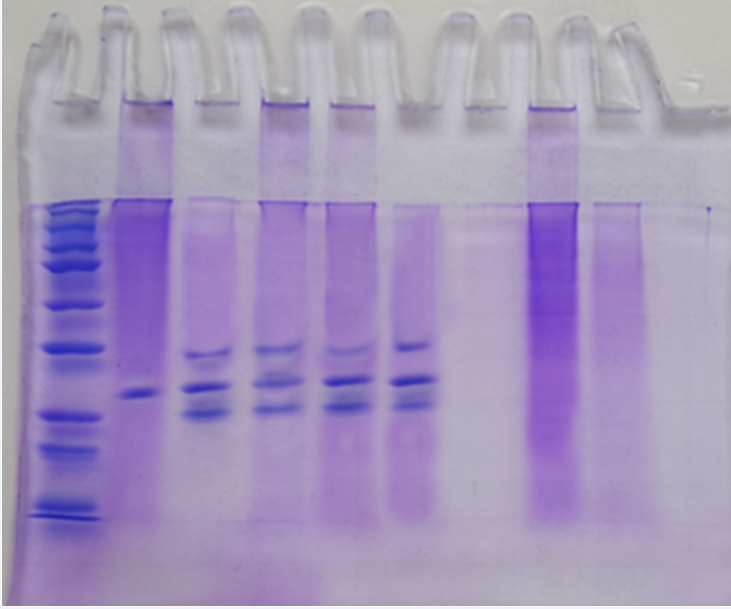

Supplement: Supplementary file 1 [file biomolecules-14-00576-s001.zip › biomolecules-2966624-supplementary.jpg]
